# Profile of Brazilian Cardiologists: An Overview of Female Leadership in Cardiology and Stress - Challenges for the Next Decade

**DOI:** 10.5935/abc.20190132

**Published:** 2019-07

**Authors:** Evandro Tinoco Mesquita, Eduardo Thadeu de Oliveira Correia, Letícia Mara dos Santos Barbetta

**Affiliations:** 1 Sociedade Brasileira de Cardiologia - Diretoria de Qualidade Assistencial, Rio de Janeiro, RJ - Brazil; 2 Universidade Federal Fluminense, Niterói, RJ - Brazil; 3 Hospital Pró-Cardíaco, Rio de Janeiro, RJ - Brazil

**Keywords:** Cardiologists, Leardership, Salaries and Fringe Benefits, Women, Gender Identity, Sexism, Prejudice

Cardiology is a medical specialty that often involves outpatient and hospital activity, leading cardiovascular care in a complex setting with aging. Increasing levels of complex decision-making and knowledge about technological resources can generate overload of activities and changes in the profile of Brazilian cardiologists, such as in their compensation, free time and stress levels. As such, medical demography studies are key to understanding the dynamics of these healthcare professionals.

In the study by Faganello et al.,^1^ the authors investigated the profile of Brazilian cardiologists in a cross-sectional study conducted in 2017, based on questionnaires sent by e-mail to fellow cardiologists of the Brazilian Society of Cardiology (SBC) who have been paying their membership fees regularly. Worth noting is that the likelihood of cardiologists with lower incomes not paying their membership fees is something that could generate bias in the study, thus influencing the results on their income. In addition to that, the study does not include anyone who has been exercising their professional activity without being members of SBC.

Of the 13,462 associates who pay their fees regularly, 70.9% are males, which is similar to the 2018 medical demographics published by the Brazilian Federal Council of Medicine, which identified 15,516 cardiology specialists, of which 70.1% were males, revealing that there is no significant difference between men and women joining SBC.^1,2^ Despite the significant difference between the percentage of female and male cardiologists, in the study concerned, women represented the largest share of the youngest age groups, which may reveal an increase in the number of women joining the cardiology community in recent years.^1^

One of the main findings of the study was the wage difference between males and females. In the higher age groups, there was a higher proportion of men (66.5% of the men reported earning more than BRL 20,000 vs. 31.2% of women), with p < 0.001, even after adjusting for working hours and age group.^1^ According to the article, a possible factor for this wage difference is the largest proportion of men working in the private sector (14% of women vs. 23.9% of men), while the proportion of women in the public and academic sectors, which offer lower pays compared to the private sector, was higher (53% of women vs. 44% of men).^1^ However, other factors should be considered.^3^ A study conducted by Douglas et al.^4^ reported that factors such as the existence of positive female models and a female-friendly specialty influence female career choices. As such, the discrepancy between the very proportion of men and women can be a factor that deter women from engaging in cardiology, in addition to the fact that there are still few women in leadership positions.

Another important aspect to be addressed, but it is not investigated in the study, is the presence of sexism in cardiology, which may be one of the factors that contribute to the lower presence of women in the specialty and their lower compensation. British studies have revealed that about 6% of the residents in the first years of specialization have experienced or witnessed sexist language, but this number rises to 15% in the last years of residence.^3^ In view of the above, approaches that seek to stop sexism at the workplace could lead to greater inflows of women in cardiology, encouraging them to move up their career ladder and take leadership positions, thus generating successful female models and better compensation profiles.

Another relevant finding of the study was the level of stress among cardiologists: 64.2% believe they have adequate levels of stress, 24.3% do not consider themselves stressed and 11.3% report stress to a great extent, which is mostly caused by poor working conditions. These data were significantly lower than the North American data, where burnout was present in 46% of cardiologists.^1^ Although this information is important, structured tools for burnout research should be implemented in future questionnaires on the prevalence of this syndrome in Brazilian cardiologists. The study also found a high percentage of cardiologists who report being careless with their own health (39.4%) and who do not perform any type of physical activity (31%), which contrasts with the US data, where only 11% of cardiologists do not perform any physical activity.^1^

**Figure 1:**
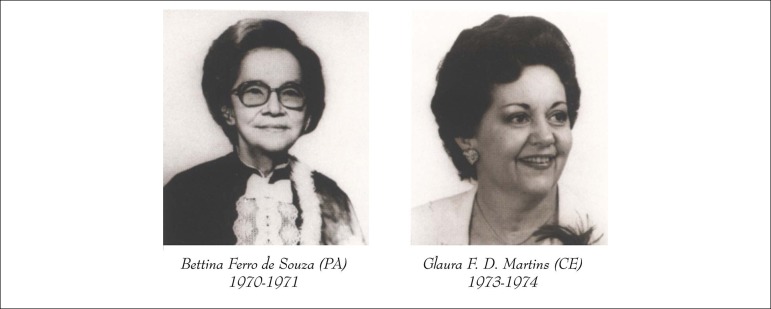
Former Presidents of SBC. Female medical leaders of cardiology in Brazil.

In a modern and increasingly egalitarian world, it is of great relevance to discuss the factors that potentially lead to wage discrepancy between genders and the low inflow of women into the cardiology community. Awareness-raising activities and female healthcare and organizations and medical societies focused on reducing sexism, encouraging the participation of women in cardiology, and the education of female leaders are key to changing this scenario. The Brazilian Society of Cardiology, since it was founded 76 years ago, it has had 57 presidents, of which only two of its main representatives were women: Dr. Bettina Ferro de Souza and Dr. Glaura F. D. Martins (Figure 1). Besides that, due to the high percentage of cardiologists who do not take good care of their own health, it is necessary to emphasize the importance of physical and mental healthcare in the medical environment and to promote improvements in working conditions.
